# Metabolic Reprogramming Underlying Brain Metastasis of Breast Cancer

**DOI:** 10.3389/fmolb.2021.791927

**Published:** 2022-01-05

**Authors:** Baoyi Liu, Xin Zhang

**Affiliations:** ^1^ Clinical Experimental Center, Jiangmen Key Laboratory of Clinical Biobanks and Translational Research, Jiangmen Central Hospital, Jiangmen, China; ^2^ Dongguan Key Laboratory of Medical Bioactive Molecular Developmental and Translational Research, Guangdong Provincial Key Laboratory of Medical Molecular Diagnostics, Guangdong Medical University, Dongguan, China; ^3^ Collaborative Innovation Center for Antitumor Active Substance Research and Development, Guangdong Medical University, Zhanjiang, China

**Keywords:** metabolic reprogramming, breast cancer, brain metastasis, metastatic cascade, drug targets

## Abstract

The development of brain metastasis is a major cause of death in patients with breast cancer, characterized by rapid progression of the disease and poor prognosis, and lack of effective treatment has existed as an unresolved issue clinically. Extensive research has shown that a variety of metabolic changes associated with cellular metastasis exist in primary breast cancer or brain metastases, therefore to elucidate metabolic characteristics at each step of the metastasis cascade will provide important clues to the efficient treatment. In this review, we discuss the changes in metabolic patterns of breast cancer cells at every step of metastasis for exploring the potential therapeutic target based on metabolic reprogramming, and provide new insights on the design and development of drugs for breast cancer brain metastasis.

## Introduction

In the process of cancer development, about 20% patients with cancer will develop brain metastasis, whose common primary tumors are lung cancer, breast cancer, colorectal cancer and melanoma. Breast cancer is second frequently to metastasize to the brain, whose incidence is estimated to be 5–20% (Achrol et al., 2019). In fact, it’s supposed to have a higher incidence since those patients without neurological symptoms were not recommend to receive brain MRI screening (Ramakrishna et al., 2014). Multiple studies have demonstrated that the prevalence of brain metastases of breast cancer is increasing, and the patients have rapid progression of the disease and poor prognosis (Martin et al., 2017; Kuksis et al., 2021). To date, lack of effective drugs for brain metastasis has existed as a major challenge. There are two contributing factors mainly responsible for the difficulties of treatment in brain metastases: the limitation of access of systemic drugs by several barriers in the central nervous system (CNS), and the differences of molecular characteristics as well as microenvironment between brain metastases and primary lesions. Consequently, research on the pathogenesis of brain metastasis is of great significance for finding drug targets.

**FIGURE 1:**
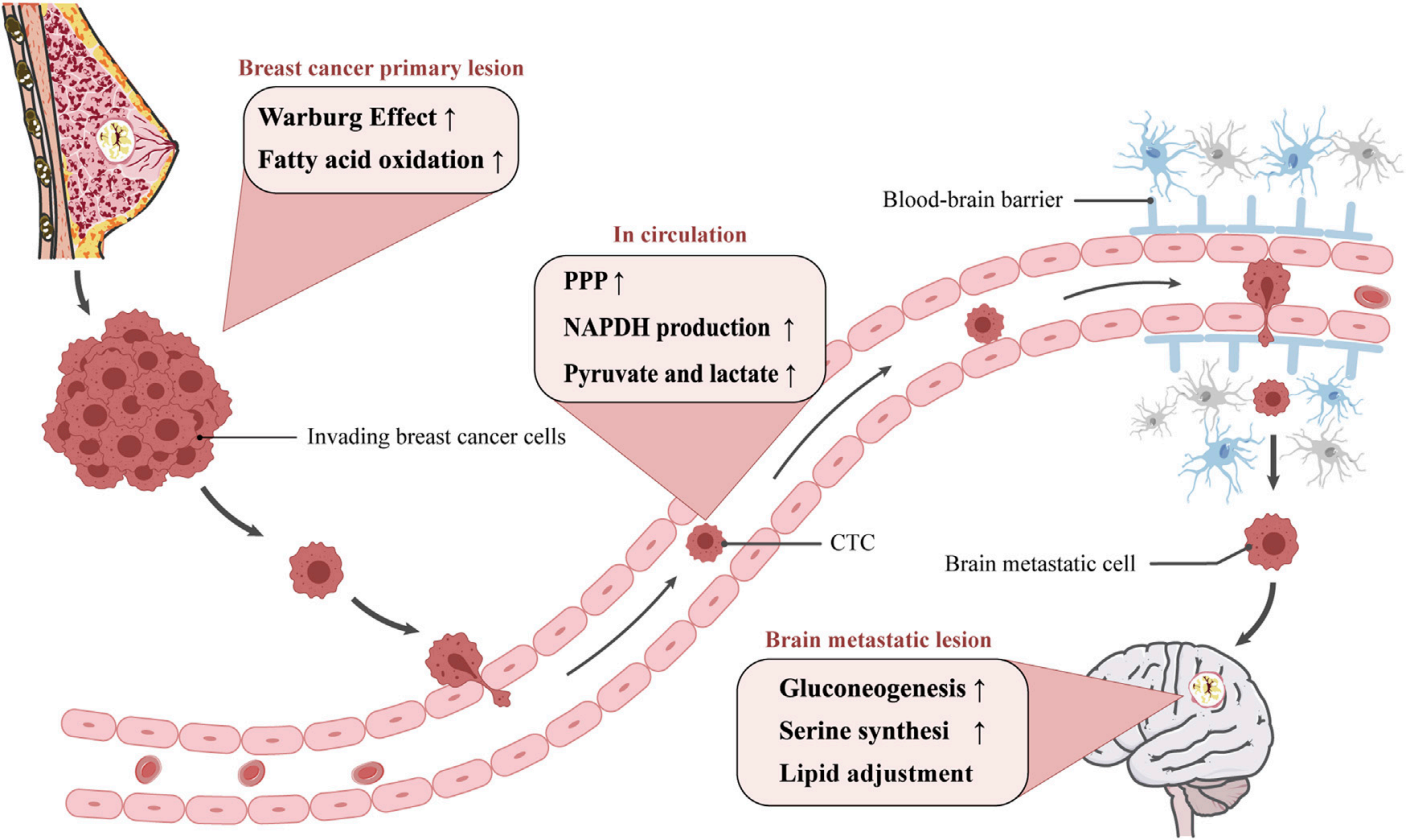
The metabolism adjustment of breast cancer cells migrating to brain. Diagram depicting potential mechanism of cellular metabolic reprogramming during different step of metastatic cascade. The metastatic route is simplified to be in the primary lesion, in circulation, and in the brain metastatic lesion. The possible metabolic reprogramming of breast cancer cells at various stages is outlined in the pink boxes.

Metastasis establishment is a dynamic process, referred to as invasion-metastatic cascade. As a first step, cancer cells invade locally then intravasate into nearby blood and lymphatic vessels, followed by transit through the lymphatic and hematogenous systems, and finally cancer cells in the circulation manage to extravasate from vessels then grow into metastatic lesions (Chaffer and Weinberg, 2011; Valastyan and Weinberg, 2011). Actually, brain metastatic cells differ from primary breast cancer cells in a number of important ways. Brain metastatic cells need to acquire certain metastatic trails to escape from apoptosis, migrate to the brain, break through the blood-brain barrier and able to survive and grow in the brain, therefore metabolic changes have a pivotal role in metastasis formation (Bergers and Fendt, 2021). This review set out to mainly discuss metabolic traits during different step of metastatic cascade (Figure 1) and explore potential metabolism-based therapeutic strategies.

## Metabolic Reprogramming in Breast Cancer With Brain Metastasis

### Metabolic Reprogramming in Invading Breast Cancer Cells

The first step of metastatic cascade is invasion of nearby tissues by cancer cells. Cancer cells of primary lesion probably go through epithelial-mesenchymal transition (EMT) to become motile and invasive. Although no definite pattern of metabolic changes has been identified that discriminates invasive from noninvasive cancer, several studied have shown that breast cancer cells undergoing EMT display enhanced glycolysis and lipid metabolism, and this metabolic change may help tumor cells to obtain local invasion ability.

#### Warburg Effect May Induce EMT to Promote Tumor Metastasis

The energy metabolism of most tumor cells shows notable differences compared with normal cells. Normal cells utilize the capacity of mitochondrial oxidative phosphorylation, while most tumor cells utilize aerobic glycolysis, which has been termed the Warburg effect (Warburg et al., 1927). The change of energy pattern is linked to the metastasis of breast cancer. On the one hand, abnormal expression of glucose metabolism enzymes like hexokinase (HK) and Pyruvate kinase M2 (PKM2) was found in primary breast cancer tissues. When their expressions are down-regulated, the glycolytic activity and lactate expression will be decreased, thereby preventing tumor growth and development (Zhang et al., 2019; Yu et al., 2020). Moreover, Fructose-1,6-bisphosphatases (FBP) has been found to be inhibited by Snail-G9a-Dnmt1 complex in basal-like breast cancer (BLBC), and Snail is critical for E-cadherin promoter silencing (Dong et al., 2013).

On the other hand, there is evidence that certain metabolites of aerobic glycolysis enable cancer cells migration by participating in modulating activity of signaling pathways, for example, increased pyruvate and lactate metabolism directly stimulate the invasion and migration potential of cancer cells. The acidification of the microenvironment caused by the increased lactate, an end-product of glycolysis, entered the endothelial cells through lactate transporters such as MCT1 which activated transcription factor NF-κB to promote cancer cell migration. NF-κB is involved in the degradation of extracellular matrix (ECM) and angiogenesis, and related to the inducement of EMT (Payen et al., 2016; Payen et al., 2017). In addition, the formation of methylglyoxal, through aerobic glycolysis, resulted in elevated nuclear YAP (Nokin et al., 2016), which is a key transcriptional co-activator for regulating tumor growth and invasion (Lundo et al., 2020). Thus, presumably enhanced aerobic glycolysis directly or indirectly contribute to promote tumor invasion and migration by driving ECM degradation and EMT program.

#### Asparagine and Glutamine Metabolism Co-Regulate Tumor Cell Metastasis

Asparagine and glutamine are likely to work in concert to drive tumor growth and metastasis by regulating cell survival, growth, and EMT regulatory pathways (Luo et al., 2018). In a mouse model of breast cancer, the asparagine content in the protein driving the EMT is selectively increased, suggesting that the asparagine in part governs metastasis by modulating the EMT program. Of note, aspartic acid can be converted into asparagine by asparagine synthetase (ASNS), and reduction of asparagine by knockdown of ASNS or treatment with bacterial l-asparaginase reduces breast cancer invasion, circulating tumor cells (CTC), and metastasis without affecting primary tumor growth (Knott et al., 2018).

Both glutamine and glucose are crucial fuel for cancer cells and generate biosynthetic intermediates for the synthesis of macromolecules (Vander Heiden and DeBerardinis, 2017). A study has revealed that the conversion of glutamine to glutamate in invasive breast cancer cells activated up-regulation of MT1-MMP, leading to basement membrane disruption and cell invasiveness (Dornier et al., 2017). It was surprising that as extracellular glutamine levels declined, tumor cells became asparagine-dependent for proliferation and protein synthesis (Pavlova et al., 2018).

Together, the studies described above support the notion that asparagine and glutamine jointly regulate the survival, growth, and metastasis of tumor cells, but it remains elusive as to how they affect the EMT-related protein during the invasion stage.

#### Fatty Acid Metabolism Provides Energy for Tumor Cell Invasion

To some extent, lipid metabolic adjustment is correlated with the occurrence and metastasis of breast cancer. For example, fatty acids probably can be oxidized to fuel for tumor cell invasion. Previous studied has documented compared with the adipocytes cultured alone, the fatty acid oxidation of breast cancer cells was significantly increased when cocultured with adipocytes (Wang et al., 2017). If co-cultivated cells were injected into mice from their tail vein, the metastasis ability of these cancer cells in mice was significantly enhanced (Dirat et al., 2011). Besides, breast cancer cells down-regulated the mitochondrial protein LACTB by ZEB1, resulting in increased phospholipid metabolism, cancer cell proliferation, and EMT inducement (Keckesova et al., 2017). Intriguingly, the overexpression of human epidermal growth factor receptor 2 (HER2) was observed to indirectly activated ZEB1 to promote cell migration (Zeng et al., 2019).

Although the potential mechanistic link between fatty acid metabolism and metastasis formation has not been disclosed, studies have confirmed that CD36, which is highly expressed in a variety of metastatic cancer cells, regulates fatty acid metabolism and plays an important role in the invasion and metastasis of various types of tumors (Pascual et al., 2017). CD36 is a transmembrane protein that promotes fatty acid entry into cells. The secretion of breast-associated adipocytes induced CD36 expression, thereby enhancing the ability of breast cancer cells to uptake fatty acid and their invasiveness *in vitro*. In line with this, inhibition of CD36 reduced lipid droplet accumulation and weakened the aggressiveness of breast cancer cell lines (Zaoui et al., 2019). Thus, CD36 may serve as an important regulatory factor mediating reprogramming of fatty acid metabolism in breast cancer cells.

As a precursor of lipid synthesis and the end product of fatty acid β-oxidation, changes in acetyl-CoA levels can also affect lipid anabolism in tumor cells. Phenotypically, increased invasiveness of breast cancer cells was induced by the upregulation of acetyl-CoA (Schug et al., 2015). Mechanistically, leptin and TGF-β1 inhibited the lipogenic enzyme acetyl-CoA carboxylase (ACC) 1 via TAK-AMPK pathway, resulting in the accumulation of acetyl-CoA, which activated the EMT program through Smad2 transcription factor acetylation, then induced breast cancer cell invasion (Rios Garcia et al., 2017). Taken together, these findings indicated that fatty acid and acetate are used as alternative nutritional source by metastasizing cancer cells.

### Metabolic Reprogramming in Circulating Breast Cancer Cells

After the tumor cells detach from the primary focus, they infiltrate into the blood vessels and migrate along with the blood circulation system. On account of exposure to various stresses in new environments, such as increased oxidative stress and attack by immune cells, most CTC undergo apoptosis or phagocytosis in circulation, and only a few cells manage to escape and develop into metastatic tumor, which referred to as anoikis (Frisch and Francis, 1994). Therefore, cancer cells need to strengthen their antioxidant defense in the circulation through certain mechanisms such as metabolic remodeling to avoid cell death result from matrix detachment. Several studies have shown that the protection of CTC from oxidative damage mainly depends on nicotinamide adenine dinucleotide phosphate (NADPH) produced by pentose phosphate pathway (PPP) to avoid the hazard of reactive oxygen species (ROS).

#### Upregulation of PPP Support the Survival of Detached Metastasizing Cells

Once tumor cells enter the circulatory system, they will produce reduction products NADPH and glutathione (GSH) through PPP pathway, endowing cells with stronger antioxidant capacity and eliminating ROS, thus reducing cell anoikis (Schafer et al., 2009). In the circulatory system, cells may induce metabolic changes through corresponding gene changes. For instance, a study has reported that the glucose uptake and ATP production in normal mammary epithelial cells separated from ECM were significantly reduced. Interestingly, when the ErbB2 signal was activated, it could restore glucose uptake and ATP production. Additionally, over-expression of ErbB2 also activated the PPP, which increased NADPH production to protect CTC from oxidative stress (Schafer et al., 2009).

#### Pyruvate and Lactate Metabolism Protect CTC Against Oxidative Damage

The metabolism of pyruvate and lactate is conducive to the resistance of matrix detachment-induced cells death. There was supporting evidence that serum pyruvate concentration in patients with advanced metastatic breast cancer was higher than that in patients with localized early breast cancer (Jobard et al., 2014). When pyruvate carboxylase (PC) transcription was down-regulated, the acetone-to-oxaloacetic acid pathway was blocked, resulted in a decreased ratio of NADPH/NADP+ and GSH/GSSG, leading to increased oxidative stress (Wilmanski et al., 2017). The high concentration of pyruvate and lactate in the blood possibly promoted the hypoxic response and maintain the activity of CTC and cell clusters through the hypoxia-inducible factor 1α (HIF-1α) (Vande Voorde et al., 2019). The studies described above suggested that pyruvate can act as an extracellular antioxidant to facilitate cells survive by enhancing the resistance of CTC to oxidative stress, and lactate drives the PPP pathway to produce NADPH(Ying et al., 2021). In conclusion, antioxidant defense is essential to avoid tumor cell death in circulation. Treatment based on metabolic reprogramming in CTC, in combination with surgical treatment of the primary tumor, may better reduce the recurrence and metastasis of cancer.

### Metabolic Reprogramming in Metastatic Breast Cancer Cells During Brain Colonization

Although upregulation of expression level of the key glycolytic enzyme HK2 gene and glucose transporter 3 was found in brain metastases (Palmieri et al., 2009; Kuo et al., 2019), increased glycolytic activity does not appear to be a metabolic feature of brain metastases from breast cancer. This was exemplified in a metabolic study of breast cancer brain metastases, which has found that the metastatic cells can rely on gluconeogenesis and the oxidation of glutamine and branched chain amino acids to satisfy energy requirements, thus evolving their ability to survive and proliferate independent of glucose (Chen et al., 2015).

#### Enhancement of FBP-Dependent Gluconeogenesis Found in Brain Metastases

FBP is one of the key enzymes in the gluconeogenesis pathway. Expression levels of FBP and glycogen were elevated in brain metastases compared to primary breast tumors in patients. In BLBC, silencing FBP could induce glycolysis, leading to an increase in glucose uptake and a decrease in the activity of metastatic cells. It could also suppress oxygen consumption by inhibiting mitochondrial complex I to activate oxidation products (Chen et al., 2015). In contrast, FBP was inhibited in invading breast cancer cells (Dong et al., 2013). Nevertheless, FBPS and glycogen are indispensable substances for normal metabolism of the human, thereby they may be difficult to serve as drug targets in brain metastases.

#### Upregulation of Serine Induced by PHGDH in Brain Metastases

Serine is a kind of non-essential amino acid. In addition to food supply, serine can be produced by cells *in vivo* via the serine synthesis pathway. A functional genomics study revealed that SSP is essential for the development of breast cancer (Possemato et al., 2011). The Phosphoglycerate dehydrogenase-catalyzed process is the first step in the serine biosynthesis pathway. Up-regulation of phosphoglycerate dehydrogenase (PHGDH) protein expression may occur in approximately 70% of ER-negative breast cancer tissues (Possemato et al., 2011). The expression of PHGDH in breast cancer brain metastases was significantly higher than other metastatic colonization such as lung, liver, and ovary. After suppression of PHGDH by genetic silencing or inhibitor such as PH-755, metastatic capacity of cancer cells was impaired and overall survival of the mice was improved due to a decrease in serine content in the brain, yet the growth of extracranial tumors (metastases such as lung and liver) was not affected (Ngo et al., 2020).

#### Breast Cancer Cells Display GABAergic Properties in the Brain Microenvironment

A study in brain metastasis has shown that breast-to-brain metastatic tissue and cells displayed a GABAergic phenotype similar to that of neuronal cells. By using γ-aminobutyric acid (GABA) as an oncometabolite, brain metastases catabolize GABA into succinate to promote the tricarboxylic acid (TCA) cycle, thus indirectly increasing cell proliferation (Neman et al., 2014). What’s more, brain interstitial space contains high levels of glutamine, which is an important precursor of the neurotransmitters glutamate and GABA (Chen et al., 2015). Above evidence suggested that glutamine serve as energy substrates in the brain microenvironment, but it cannot be used as a therapeutic target due to little is known about the concrete mechanism of utilization.

#### Lipid Adjustment Is Important for Brain Colonization

Jin et al. pointed out that adjustment in lipid metabolism were necessary in breast cancer brain metastasis, and treatment on lipid metabolism of breast cancer cells might be beneficial to curb brain metastasis development (Jin et al., 2020). Fatty acid binding protein (FABP 7) is a brain-specific intracellular lipid-binding protein, and its overexpression was shown to coincide with the low survival rate of patients with HER2-enriched breast cancer and the increased incidence of brain metastasis. FABP7 is involved in the metabolic reprogramming of cancer cells, supporting the glycolytic phenotype and the storage of lipid droplets, thus enabling them to grow in the unique environment of the brain. What was surprising was that up-regulation of FABP7 was not detected in the primary breast cancer, which supported the potential function of FABP7 on the brain viability of breast cancer cells (Cordero et al., 2019).

Fatty acid synthase (FASN) is likely to be another potential target for inhibiting brain metastasis of breast cancer (Menendez and Lupu, 2017). FASN is the key enzyme in fatty acid biosynthesis, and its expression is necessary for lipid synthesis and maintenance of palmitate level, especially in the environment of exogenous lipid deficiency, such as the brain. Additionally, the genetic and pharmacological effects of FASN abolished the growth of brain metastases, further demonstrating that fatty acid synthesis is crucial for the growth of metastatic tumor in the brain (Ferraro et al., 2021).

Brain metastases could also use acetate as a compensatory source of carbon to support *de novo* lipid synthesis (Mashimo et al., 2014). Acetate oxidation has been verified in primary and metastatic mouse brain tumors and patients with brain metastases, and this process could be performed simultaneously with glucose oxidation. Of interest, glucose contributed less than 50% of the acetyl-CoA pool (Maher et al., 2012), suggesting that tumors utilized additional substrates, thus glucose may be a minor source of acetyl-CoA in the TCA cycle (Mashimo et al., 2014).

## Targeting Metabolism to Treat Breast Cancer With Brain Metastasis

The existence of human blood-brain barrier greatly hinders the killing effect of chemotherapeutic drugs on brain tumors. Therefore, surgery and radiotherapy remain the main treatment for brain metastases. Over the past decades, molecular targeted drugs against a variety of key enzymes of glycolysis, tricarboxylic acid cycle, lipid metabolism, amino acid metabolism and other metabolic pathways are being developing, and some of the drugs have involved in clinical trials, showing good clinical application prospects. Metastatic breast cancer cells display metabolic flexibility, that is, cancer cells can use different metabolites to meet the different metabolic requirements in specific steps of the metastasis cascade, so targeting metabolism reprogramming tend to be one of the effective strategies for the treatment of brain metastases from breast cancer.

### Targeting Metabolic Enzyme of Brain Metastatic Cancer Cell

The Warburg effect endows breast cancer cells with the ability to invade by inducing EMT, thereby targeting glycolysis metabolism probably is a promising therapeutic strategy. For example, the HK2 inhibitor, 2- deoxy -D- glucose (2-DG), inhibited tumor metastasis by inducing a change in the metabolic pattern of oxidative phosphorylation and reducing the production of lactate. Yet, it showed no obvious therapeutic effect on mouse xenografts and patients (Zhang et al., 2019).

Altering the acidic environment presumably is another extremely effective strategy to block metastasis. There is abundant evidence that accumulation of the metabolic byproduct lactate and extracellular acidification exacerbates tumor cell proliferation, metastasis, and angiogenesis. The acidic environment created by lactate accumulation promotes the degradation of ECM, mainly due to PH decline could stimulate the secretion and activation of hydrolases, including cathepsins and MMP-9 (Payen et al., 2016). MMP-2 and MMP-9 are also correlated with the development of breast cancer metastasis (Li et al., 2017). Furthermore, extracellular acidification mediates immunosuppression and reinforces tumor immune escape. Brown et al. observed that breast cancer cell-derived lactic acid activated G-protein-coupled receptor (GPR) 81 in dendritic cells and prevented the presentation of tumor-specific antigens to other immune cells (Brown et al., 2020). Chen et al. reported that lactate activates macrophage GPR132 and thus promotes lung metastasis of breast cancer (Chen et al., 2017).

What’s more, enzymes involved in lipid metabolism may also be suitable targets for preventing the formation of brain metastases, such as FASN, which is a promising target for the treatment of brain metastasis originated from breast cancer (Kingwell, 2021). Moreover, PHGDH inhibitors that interfere with serine synthesis in the brain may also contribute to the treatment of brain metastases (Ngo et al., 2020).

### Blocking Signaling Pathways for Metabolic Regulation of Brain Metastases

Existing research recognized the critical role played by Notch signaling in regulating metabolism during brain metastasis of breast. High expression of IL-1β in brain metastases activated the expression of peripheral astrocyte JAG1, and the subsequent their interaction leaded to the activation of Notch pathway and promotes the growth of brain metastasis of breast cancer stem cell-like cells. On the contrary, the inhibition of Notch signal significantly prevents the occurrence of brain metastasis (McGowan et al., 2011; Xing et al., 2013).

PI3K/AKT/mTOR pathway is an intracellular signaling pathway significant for cell metabolism involved in cancer metastasis. EGFR signal activated PI3K/AKT/mTOR pathway, and up-regulated HIF-1α-mediated enhancement of glucose uptake and glycolysis-related gene expression, which may be driven by or in synergy with c-MYC to promote tumor proliferation (Masui et al., 2014). Several studies have documented that immune checkpoint inhibitors such as PD-1 could decrease mTOR activity and thus inhibited the Warburg effect (Chang et al., 2015). And it has been reported that the mTOR inhibitor rapamycin could reduce the expression of FASN in breast cancer cells (Yan et al., 2014).

According to these data, we can infer that Notch signaling and mTOR signaling axis are key players in modulating cellular metabolism and tumor growth during brain metastasis. But since the signaling pathways are interrelated with each other, it may be necessary to target multiple pathways simultaneously to obtain effective antitumor activity.

## Conclusion

In recent years, attention has been drawn to the fact that energy metabolism reprogramming is crucial for primary tumor growth, invasion and metastasis, however, the metabolic interaction has not been elucidated. Molecular subtypes may be one of the factors affecting metabolic energy supply. Gene expression analysis has identified different molecular subtypes of breast cancer, including Luminal A, Luminal B, HER2-enriched and the basal-like subtype (largely overlaps with triple negative breast cancers, TNBC), and each subtype of breast cancer has a unique metastasis pattern. In particular, patients with HER2-enriched and TNBC are the most likely to develop brain metastasis (Kennecke et al., 2010; Kuksis et al., 2021). Luminal subtypes are found to exhibit reverse-Warburg/null phenotypes, in contrast, TNBC preferentially utilize the glycolysis energy (Choi et al., 2013). Nonetheless, it remains unclear whether different subtypes of cancer cells affect the selection of metastasis sites by adopting diverse metabolic strategies (Gandhi and Das, 2019). Another contributing factor of metabolic differences is tumor microenvironment. Tumor metabolism is environmentally dependent, and the availability of nutrients in the environment indirectly affects metabolic changes in cancer cells. The breast is mainly composed of breast and fat, of which fat accounts for about 90%. By contrast, the brain contains high levels of glutamine and branched chain amino acids, which can serve as alternative energy substrates to power metastasizing cells colonized in the brain.

Although tremendous progress has been made in the research on the mechanism of metabolic therapy, the main challenge faced by drug development are as followed. First, due to pharmacokinetic constraints of blood-brain barrier, most of the existing targeted drugs cannot reach the brain metastases successfully. Second, those drugs targeting metabolic pathways show significant side effects, which presumably on account of the metabolic heterogeneity in tumors and metabolic compensation pathways. Therefore, cancer metabolism research should take into consideration genetic factors, the cell interaction in the micro-environment, the influence of diet and microorganisms on tumor metabolism preference. Last but not least, there still lack of effective biomarkers for the prediction of metastatic risk and the evaluation of metabolic therapies, making it difficult to determine the optimal therapeutic window for drugs in patients. In summary, energy metabolism reprogramming is a key feature in the occurrence and development of tumors. With the deepening understanding on tumor metabolic mechanisms, it will better help people to discover metabolism-based drug to treat breast cancer with brain metastasis.
